# Collectanea

**Published:** 1829-04

**Authors:** 


### COLLECTANEA.

Floriferis ut apes in saltibus omnia libant,

Omnia nos, itidem, depascimur aurea dicta.

### PHYSIOLOGY.

*Functions of the Intestinal Canal and Liver in the Human Fœtus.*—A very interesting paper on the above subject was lately read at the Royal Society, by Dr. Lee. From the circumstances of the early development of the liver and intestines of the fœtus, of the copious supply of blood which they receive, and of the great space which they occupy in the abdomen, the author was led to the conclusion that they performed some important functions in the fœtal economy. Although no nutritive matter can be furnished by the mouth, yet the contents of different portions of the alimentary canal were found, both in appearance and chemical composition, to have a striking analogy to those of the same parts of the canal in the adult, where the processes of assimilation and absorption are performed. A semifluid matter, possessing all the characters of albumen, is found closely adhering to the inner walls of the small intestine, and is more especially abundant around the papillary projection, through which the common duct of the liver opens into the duodenum, and diminishes in quantity as we trace it towards the termination of the ileum. The great intestines are generally distended with a dark green homogeneous fluid, containing no albumen, and apparently excrementitious. No albumen can be detected in the contents of the stomach: hence the author infers that an absorption of some nutritious substance (which he brings forward several arguments to show must be derived from the liver,) takes place from the intestinal canal in the latter months of gestation. He states that in two instances he detected the presence of a substance similar to that which he had found in the duodenum, in the hepatic duct itself. Hence he is led to the conclusion that the function of the liver in the fœtus is not confined to the separation of excrementitious matter from the blood, but that it supplies materials subservient to nutrition. That the substances existing in the intestines of the fœtus are not derived from the mouth, is proved by these being equally found in encephalous children, or where the œsophagus is impervious, as where no such malformation had existed.

A note is subjoined to this paper by Dr. Prout, giving an account of the mode by which he ascertained the chemical character of the substance referred to in his examination. The paper is accompanied by drawings of the intestinal tube in the fœtus.—*Philosophical Magazine.*

---

*Experiments on the Velocity of the Circulatory Motion of the Blood, and on the Quickness with which the Secretions are performed.* By E. Hering, Professor at the Veterinary School of Stutgard.

These experiments, to the number of eighteen, were made on horses. There was injected, or rather infused, into the jugular vein, a solution of hydrocyanate of potash and iron. To introduce the liquid he employed a glass tube, of the size of a writing quill, capable of being closed by means of a stop-cock, and surmounted by a brass funnel, holding two ounces of liquid. The tube being introduced into the vein, the funnel was filled, and the cock opened, to be shut again the moment the liquid had passed into the vein. In this manner the access of air was prevented. After some time a vein was opened in another part of the body, and the blood examined by chemical reagents. The animals were killed some time after, and the traces of the liquid introduced was sought for in the secreting organs and their products.

The second series of experiments is to form the subject of a separate memoir.

As a reagent for discovering the hydrocyanate of iron in the blood, the author preferred the sulphate of iron to the sulphate of copper, or the hydrochlorate of iron. To form a blue precipitate with this salt, it was only necessary to add a little hydrochloric acid. The hydrocyanate of potash and iron was also recognised, although existing only in the proportion of one to 20,000 in the serum of the blood. The small quantities of hydrocyanate not being discoverable in the coloured blood, it was necessary to let it rest from twenty-four to forty-eight hours, to have a clear and limpid serum. Some drops were let fall on white paper, and there were afterwards added a few drops of a solution of sulphate of iron, (one grain in three ounces of distilled water:) a drop of concentrated hydrochloric acid, which was added in the last place, instantly betrayed the presence of the hydrocyanate. This procedure is equally applicable to the solid parts that are submitted to examination.

1st. A solution of hydrocyanate of potash and iron, introduced into the jugular vein of the horse, runs the course of the circulation, and arrives in the jugular vein of the opposite side, *in an interval of from twenty to twenty-*

*five seconds*, or from twenty-five to thirty. It arrives in from twenty-three to thirty seconds in the external thoracic vein of the opposite side, in twenty seconds at the vena saphena major, in from fifteen to twenty seconds in the mesenteric artery, and in from twenty to twenty-six seconds, in the maxillary artery; lastly, in from twenty to twenty-five, and from twenty-five to thirty seconds, in the metatarsal artery, always on the opposite side to the place of injection.

If the liquid introduced by this injection is moved by the same means as the blood, the velocity of the motion must be the same in both. It appears that the velocity of this motion is not increased in the ratio of the number of pulsations of the heart; for in a horse in which the pulse was sixty in the minute, and in two others in which it was from thirty-six to forty-four, and from forty-eight to fifty-two, the results were the same. Yet in another, in which the pulse was from thirty to forty-four, the circulation was found to be some seconds slower.

2d. The hydrocyanate of potash and iron is promptly secreted by the serous membranes, but in small quantities; and this in the direct ratio of their distance from the heart. Thus, the secretion commences by the internal surface of the pericardium, where it is also the most abundant; it then takes place in the pleura, the peritoneum, and lastly in the articular capsules. The cerebral cavities were opened only in a few cases; and there was never found any trace in them of the saline solution injected. In the other serous cavities, the presence of this solution was discovered two, three, four, seven, and fifteen minutes after injection. These moments were also those when the animal ceased to give symptoms of life.

3d. The mucous membranes secrete the injected solution less quickly than the serous. A few minutes are, however, sufficient to discover the foreign principle at their free surface; and soon after it is found at their other surface. The mucous membrane of the right half of the stomach secretes more promptly than that of the intestine, and the latter more quickly than the surface of the lungs. Secretion is much slower in the genito-urinary surface; what was found of the solution in the urinary passages came only from the kidneys.

The mucous surfaces covered with an epithelium (as the walls of the mouth, the pharynx, the left half of the stomach, in the horse,) gave no traces of secretion of the saline solution injected.

4th. The liver, the spleen, the thyroid gland, &c. allow the presence of the hydrocyanate to be detected but with difficulty, on account of their dark colour. The salivary glands appeared to perform a considerable part in the elimination of the foreign substance.

5th. The kidneys act also very powerfully. The reagent manifests the presence of the hydrocyanate at the expiration of one minute in the cortical and tubular parts, and in the pelvis. The passage of the urine through the ureters being rather slow, the consequence is that the bladder does not present traces of the foreign precipitate until after a pretty long interval. The small blood-vessels of the kidneys gave signs of reaction, while the large ones gave none; whence it might be concluded, either that the circulation is slower in the former, or that the hydrocyanate already commences beforehand to separate from the blood.

6th. In the lungs this salt is not so difficultly discovered as might have been presumed.

7th. The saline solution adheres in some cases to the walls of the blood-vessels, and is then easily discovered by the reagents; more frequently it does not adhere to them. Sometimes it adheres in some of them, and not in others. The cause of this difference is unknown.

8th. The shortest time which the solution takes to reach the thoracic duct is still undetermined. A minute sufficed in one case, and from two to five minutes in others. It is only discovered a little later in the lymphatic ganglia, although it already occurs in the thoracic duct. It therefore would appear, says the author, that there is a direct communication between the arteries and lymphatic vessels.

9th. The foreign substance introduced into the blood is quickly ejected by the secreting organs, especially by the kidneys. In from five to eight hours there no longer remained any traces of it in the products of the secretions; and in twenty-four hours all traces had disappeared, even from the solid parts.

10th. Lastly, it appears from these experiments, that the hydrocyanate of potash and iron may be mixed with the blood without inconvenience to the animal. A solution of indigo has not the same advantage. A solution of sulphate of iron injected into the blood coagulates it, and speedily causes death.—*Zeitschrift für Physiol.* t. ii. p. 85.)

### PATHOLOGY.

*Case of Pulmonary Apoplexy.* By Dr. PINGRENON, Surgeon of the Royal Artillery.

M. Del—, residing at Fére, of the middle size, rather fat, aged twenty-nine, was several years subject to affections of the stomach, for which he occasionally applied leeches to the anus. On the 31st August, 1828, after six months of unremitting occupations at the desk, he was seized with a slight return of his old complaint, for which he could assign no cause, and for the removal of which he adopted only a cooling regimen. But, as his health daily grew worse, he sent for Dr. LEFRANC on the 2d of last September, who found the patient confined to his room: his face was pale, his eyes swollen and watery, and, on rising to reply to the questions of his physician, he complained of a fixed pain at the middle of the sternum, attended with heat and some difficulty of breathing, and suddenly exclaimed that "the pain was quite gone." His face became immediately livid, his lips were covered with a bloody froth, and he fell down and expired before any assistance could be rendered.

On immediately inspecting the exterior of the body, livid stripes were noticed at the anterior and superior parts of the thorax, which diappeared the next morning.

Drs. Pingrenon and Lefranc opened the body forty-eight hours after death, and remarked the following particulars:

The body had lain, since the patient's decease, on the back, with the head rather elevated: there was some bloody froth on the mouth; the face and scalp were livid and emphysematous; the sides of the head, front of the neck, upper and anterior parts of the thorax, were all, as far downwards as the mammæ, more or less emphysematous, swollen, and of a dark slate colour, which gradually disappeared towards the abdomen. The belly was somewhat distended with flatus. The back of the trunk, &c. presented no unusual appearance.

The integuments of the head were very loose. The dura mater was not vascular, but somewhat of a dull colour. The lateral and longitudinal sinuses were empty; as were also the internal carotids, which had been divided to extract the brain. On the arachnoid membrane and pia mater there were some dark red patches, and some minute black coagula between a few of the upper and middle convolutions of the brain. The cortical and medullary substances had no uncommon appearance. The lateral ventricles were empty. The choroid plexuses were injected with darkish blood.

The cellular membrane of the anterior surface of the thorax was very dark and vascular, and crepitated when pressed together with the fingers. The muscles were of a pale red, and rather soft. The lungs were much distended, and in one spot adhered slightly to the pleura costalis: towards their summit they were of a deep violet colour, and crepitated; in other parts they were gorged with black blood, which, notwithstanding many incisions, was with difficulty squeezed out. On washing the divided portions, the parenchyma was found of a dark colour, owing to the cellular membrane containing within its cells a quantity of coagulated blood, by which the lungs were rendered impervious and apparently hepatized. The pericardium was sound, but contained four drachms of bloody serum. The heart was pale and soft; the auricles and ventricles were empty.

The stomach was distended with air, and there were dark red spots in many parts of its external surface. The great omentum was much injected, and drawn back towards the great curvature of the stomach, the mucous membrane of which was soft and deeply corrugated, and stained with spots of a blackish description near the cardia, along the small curvature, and towards the pylorus, which, after washing, retained a mottled appearance. In the small intestines slight traces of inflammation were visible, and the air they contained was rather fetid. The liver was small, and its concave surface was of a deep violet colour. The gall bladder was full of yellow bile, which was beginning to transude. The other viscera were not examined.

Pulmonary apoplexy is one of those diseases whose etiology and gradual development are very imperfectly understood. The suddenness with which it attacks those who appear to be in perfect health; the rapidly fatal consequences which always ensue; the extraordinary traces of alteration which mark its occurrence, afford ample scope for study, and should engage us not to omit any facts which are in the least calculated to elucidate this obscure department of pathology. It is with this view that we have detailed the above case, on which we forbear making any reflections, trusting that it may eventually elicit some useful communications from others.

Dr. Pingrenon considers that the pulmonary congestion might, in this instance, be owing to sympathy between the stomach and the lungs.—*Revue de Médicine, Françoise et Etrangere.*

---

*Case of Pneumo-Thorax; with an Account of an Operation performed for its Relief, the Effects of the Operation, and the Appearances on Dissection.* By Dr. James Johnson.

Mr. Cornish, surgeon, residing in Milner place, near the Coburg Theatre and aged about twenty-seven or twenty-eight years, became affected with dyspnœa and symptoms of thoracic inflammation, about the latter end of November or beginning of December last, which he neglected for many days, and continued to pursue his avocations in the three branches of the profession. About the 15th or 16th of the same month, he was accidentally seen by Mr. COOKE, (an intelligent practitioner, of Bridge street, Lambeth,) who strenuously recommended sanguineous depletion, confinement to the house, and the other items of the antiphlogistic treatment. It was with difficulty he could be persuaded to take to his room, but he was too ill to go on longer with his practice.

On the 19th or 20th of December, Dr. JOHNSON was requested to see Mr. Cornish, and found him in the following condition: The patient was of the scrofulous character. He was lying on a sofa on his right side, breathing with considerable difficulty, and frequently coughing; the expectoration was scanty, and extremely tenacious, but without any purulency; the pulse was 130, sharp and wiry; skin not very hot nor dry; tongue moist, thirst moderate; right cheek flushed; urine high coloured and scanty. He complained of great difficulty of breathing, had pain in the centre of the chest, and could only lie on the right side. On uncovering the thorax, the muscles of respiration were seen in violent action, but the breathing was principally carried on by the diaphragm. There was no perceptible difference in the size of the two sides of the chest, but a very remarkable difference in the sound emitted on percussion: the *left* side sounded louder than natural, the *right* sounded considerably duller than natural. On applying the ear to the left side, which sounded so well, little or no respiration could be heard: on listening to the right side, which sounded so dull, the respiration was very loud, and accompanied with much wheezing. The heart was felt beating rather to the right of the middle of the sternum, and no trace of it could be felt in the left side.

These phenomena appeared to Dr. Johnson to be very unfavorable; but, as inflammatory action was still unequivocal in the case, Dr. J. advised Mr. Cooke (who kindly and zealously attended his afflicted neighbour till the last) to take away more blood, both generally and locally. Digitalis, colchicum, and antimony were also given in powerful doses, with the view of making an impression on the circulation.

December 21.—The urgency of the dyspnœa was a little, and but a little, relieved by the depletion: the blood was remarkably buffed and cupped. On examining the chest this day, Dr. Johnson and Mr. Cooke found that the left side was even more sonorous than before, and the respiration there still more indistinct; the pulsation of the heart was rather farther to the right; the right side very dull on percussion, and the respiration very noisy and confused. But a most important feature of the case now attracted attention, namely, the metallic tinkling (*tintement metallique*), which was distinctly audible in the left side of the thorax, not only when the patient coughed or spoke, but even during every inspiration and expiration. Dr. Johnson had now no doubt of the existence of pneumo-thorax, as every person who put the ear to the chest heard the tinkling as plainly as himself. Upon accurate examination, the left side was found to be very sonorous back almost to the spine; which led to the conclusion that the quantity of serous, purulent, or sero-purulent effusion was very small in quantity when compared with the aeriform extravasation. What was now to be done? There was still symptoms of thoracic inflammation present; and to quell these, and promote a free expectoration, every means that could be devised was put in force.

The next five or six days were consumed in the furtherance of these indications, but with no effect in mitigating the difficulty of breathing: which, indeed, gradually increased, the pulse seldom coming under 130 in the minute, with great and distressing jactitation.

In the course of the above period several medical gentlemen saw the patient, and Dr. WALSHMAN was added in daily consultation with Dr. Johnson and Mr. Cooke.

On Monday night, the 29th December, the patient nearly expired from suffocation; and next morning (Tuesday, the 30th,) Dr. Johnson explained to the patient the nature of the case: namely, that there was an aperture in the left lung, through which air was extravasated into the left pleural cavity, which cavity also contained some fluid, the precise nature of which could not be ascertained. It was stated to Mr. Cornish that the increasing collection of air was pressing severely on the right lung, that it had already pushed the heart into the right side of the chest, and that he saw no prospect of relief but from an operation.

Dr. BLICKE, of Walthamstow, examined the patient on Tuesday morning with Dr. Johnson, and was so convinced of the existence of pneumo-thorax as the cause of the dreadful dyspnœa, that he volunteered to perform the operation. Things, however, were not sufficiently ripe for such a step, and Dr. Johnson requested the patient to name a surgeon of eminence to join in the consultation. He named Mr. LAWRENCE; and Dr. Johnson waited on Mr. L. to request his opinion on the case. Mr. Lawrence, Dr. Walshman, Mr. Cooke, Mr. J. H. Johnson, and some other medical men, met at three o'clock on that day. Mr. Lawrence accurately examined the patient: he was lying on his right side, as usual, breathing most laboriously; his countenance sunk; the pulse between 130 and 140, weak and somewhat irregular; the skin was cool, and somewhat moist; he had had no sleep for many nights. On laying bare the chest, the action of all the respiratory muscles was painful to behold, and it was evident that but a very small portion of air could be taken in at each inspiration; there was no perceptible difference in the size or shape of the two sides: the left sounded hollow throughout almost its whole extent, when Mr. Lawrence struck it; the right side emitted an extremely dull sound. The apex of the heart was now beating rather to the right of the right nipple. When Mr. Lawrence applied his ear to the left side of the thorax, he distinctly heard the metallic tinkling,* as did every one of the medical gentlemen then present. The respiration was loud and rattling in the right lung, and the expectoration muco-purulent, with streaks of blood and many black particles.

On retiring to consult, it was the opinion, not only of Mr. Lawrence, but of all the other attendants, that Mr. Cornish was so near death as to render any operation hazardous, if not unavailing: indeed, it was believed that the patient would most likely expire during such an operation as was contemplated. Mr. Lawrence, however, candidly avowed that he was satisfied of

* Some of the medical gentlemen present, and particularly Mr. J. H. JOHNSON, compared the metallic tinkling to the sounds emitted by a musical snuff-box; and this, in reality, is a more familiar as well as a more exact similitude than that which LAENNEC has employed.

the existence of pneumo-thorax, both from the confidence of Dr. Johnson's diagnosis, and from the phenomena which he had himself observed during the examination, by percussion and auscultation. He also stated it as his opinion that, under more favorable circumstances, and with the same kind of phenomena present, the operation of paracentesis thoracis would be warrantable, as the only probable mean of affording relief, whether temporary or permanent, from the difficulty of breathing resulting from the pressure of air and other fluid extravasated in the cavity of the pleura. An anodyne was prescribed.

The gentlemen separated without any resolution to meet again, as Mr. Cornish appeared to be dying; and the unfortunate patient himself expressed the most poignant disappointment that no operation was undertaken for his relief.

On that day Dr. Johnson accidentally met with Dr. BALLINGALL, of Edinburgh, Dr. PECCHIOLI, of Florence, and Mr. GUTHRIE. To these gentlemen he related the melancholy case of his medical patient; and they having expressed a wish to see him, if yet alive, Dr. Johnson solicited them to visit the patient with him. They repaired to Mr. Cornish's house at ten o'clock at night, and found the patient nearly in the same state of distress as he was at three o'clock, when Mr. Lawrence and Dr. Johnson left him. The gentlemen above mentioned recognised the auscultic phenomena which have been already detailed; and, in consequence of a most urgent solicitation, not only from the patient, but from his sisters and several relations, Mr. Guthrie agreed, in deliberate consultation with Dr. Ballingall, Dr. Picchioli, Mr. Cooke, and Dr. Johnson, to perform the operation of paracentesis thoracis, as the only measure that offered even temporary relief from the dreadful state of suffocation to which the unfortunate patient was reduced. The danger of the case was not concealed from Mr. Cornish himself, nor from any of his friends; nor was any sanguine expectation held out of recovery, but only of relief. It was stated that the operation was neither painful nor dangerous, and that it afforded the only probable chance of life that remained. The patient and friends ardently urged the operation.

An incision was made in the anterior lateral part of the left side of the chest, between the sixth and seventh ribs, and the pleura cautiously opened with the scalpel. At that instant a rush of air issued forth, with a loud hissing noise, and strong enough to extinguish several candles, had they been near the orifice. The relief was almost instantaneous. The patient turned on his back, and breathed with comparative freedom, expressing the highest sense of gratitude for the operation. He was turned round on the left side, but no fluid came from the wound. A piece of linen was placed over the orifice, and the medical gentlemen retired. The relief continued for some hours, and then the difficulty of breathing returned to a certain extent.*

Wednesday, 31st.—Mr. Guthrie, Mr. Cooke, Dr. Johnson, and several others, visited the patient at half-past twelve o'clock, and found him labouring under a considerable degree of dyspnœa, though not near so much as before the operation. It was found, on examination, that the wound was

* On returning home, at midnight, Dr. Johnson wrote a note to Mr. Lawrence, apologising for the apparent breach of etiquette, and inviting Mr. L. to see the patient next morning, stating what had been done. Mr. Lawrence, however, was not able to attend on the following day.

closed. The left side sounded nearly as sonorous as ever, and the *tintement metallique* was perfectly audible. A director was introduced into the wound, and a rush of air instantly escaped, with immediate relief, as in the first operation. A probe-pointed bistoury was passed in, and the opening in the pleura extended to the size of half an inch. The pulse had fallen to 120, the countenance was good, skin moist, expectoration more copious, and muco-purulent. On examination of the left side immediately after the escape of the air, no *tintement metallique* could be heard by any of the medical gentlemen. The patient took nourishment this day, and was seen by several medical practitioners. In the evening, when Dr. Johnson visited him, the patient was not so well, and a probe was again introduced, when air escaped with some noise. Twenty drops of laudanum were given in a saline draught, and the patient was left.

Thursday, Jan. 1st, 1829.—On visiting Mr. Cornish this day, the medical attendants were agreeably surprised to find that he had had several hours of tranquil sleep, and that for the first time during some weeks; that his breathing had been easy, the expectoration more copious, and inclining to purulency; the pulse reduced in frequency, and more expanded; the appetite good. He got out of bed this day without assistance, went to the commode, and had a natural motion. Mr. Lawrence saw the patient, and pronounced him greatly relieved. On examining the wound, a canula was pushed in, and a taper was held near it. During inspiration the canula was closed with the finger, so that no air could enter the chest; and during expiration the finger was removed from the canula, when a rush of air always escaped. This was continued until no doubt could remain as to the fact that part of the air drawn in by the mouth was thrown out of the wound at each expiration. This phenomenon, and especially the large quantity of air thus thrown out, proved that a considerable aperture of communication existed between the bronchia and the cavity of the pleura; a circumstance which greatly lessened the hopes of recovery. It was found that, since the operation, the apex of the heart beat about an inch and a half, or two inches, nearer the central line of the thorax than before. The pulsation was still, however, to the right of the line. The patient continued comfortable through the day; but Mr. Cooke was called up in the night, and found him greatly oppressed. The canula was re-introduced, and some relief followed, the wound being covered with a piece of gauze.

Friday, January 2, 1829.—It was but too evident this morning that the unfortunate patient was sinking. He had a strong convulsion early today, and about one o'clock he expired.

*Post-mortem examination.*—Mr. Cornish being of the Hebrew religion, great difficulties lay in the way of an examination post mortem; but the friends and relations of the deceased evinced much liberality, and leave was ultimately attained for dissection, though such a process was almost unprecedented among the Hebrew brethren. Previously to the examination, which was conducted by Dr. Hodgkin, and witnessed by a great number of medical men, Mr. C., a surgeon of the Hebrew religion, who had frequently visited the patient during his illness, demanded of Dr. Johnson what were the morbid appearances which he expected to find? Although this was a question which it would not be always very charitable to ask before a dissection, yet Dr. Johnson did not decline the answer, which was made in the presence not only of the above medical gentlemen, but of a number of the patient's friends:* "The disease was pronounced to be pneumo-thorax; and the morbid appearances would be a collection of air and some other fluid in the left side of the chest; collapse of the corresponding lung; aperture in the lung, capable of giving free vent to air from the lung to the cavity of the pleura; displacement of the heart; probably tubercles in the right lung."

Dr. Hodgkin then opened the body. On raising the sternum, the heart was found rather to the right of the median line of the chest. The left lung was collapsed to one fifth of its natural dimensions. The vacant space was filled with air, and about fourteen ounces of turbid serous fluid. The pleura costalis and pulmonalis presented marks of inflammation of a few weeks' standing, viz. some thin false membranes, that were easily separated by scraping with the scalpel. There were no marks of any more recent pleuritis, even in the vicinity of the wound, there being only a slight ecchymosis between the pleura and subjacent cellular tissue, for the space of a few lines around the incision. A tube was inserted into the trachea, and air blown into the lungs. The left lung expanded to a certain extent, and air was heard to bubble out. The lung was then carefully removed, and an aperture was immediately recognised at the division or cleft between the two lobes. The tube was inserted into the bronchus leading to the left lung, and Dr. Johnson blew in air. It rushed forth at the aperture, and extinguished a taper that was held near it. The aperture itself was then more accurately examined. It was circular, and capable of admitting a crowquill. It was evidently fistulous, and of several weeks' standing. It was found to communicate with a very small excavation formed by the softening down of some tuberculous masses, and into this small excavation a bronchial tube was seen to enter. Thus the communication between the trachea and the cavity of the chest was distinctly traced through a bronchial ramification, a very small tubercular excavation situated on the very surface of the lung, and an aperture through the pleura pulmonalis. The left lung presented some trifling tuberculation, but was not materially diseased.

The right lung was much more tuberculated; but the tubercles were principally in a quiescent state. There was no other disease in the chest.

Dr. Hodgkin formally declared that every iota of the diagnosis was verified by dissection, and every individual present agreed in this declaration.

In this country there is but one other case on record where the operation was performed for pure pneuma-thorax; and the operation was successful.†

* We were present when this very trying, and we think unreasonable, question was proposed to Dr. Johnson, and heard the answer given, which the subsequent examination of the body proved to be perfectly correct; highly to the credit of Dr. Johnson's pathological skill.—EDITORS.

† Mr. KELLY has related a case of this disease, in the Edinburgh Med. Comment. vol. ii. p. 427, which was produced by a suppurated tubercle that formed an opening from the bronchiæ into the pleura. The inflation extended widely over the body. The symptoms became pressing, and it was determined, under the advice of Dr. MUNRO, to evacuate the air by an opening into the chest. An opening was consequently made through the pleura. Upon withdrawing the perforator, a blast of wind issued through the canula, which blew out a lighted candle three or four times. The patient was immediately much relieved. He recovered gradually, and in three weeks ate and slept well. He enjoyed a good state of health for nearly a year: at the end of that time he was again attacked with pulmonic disease, and he died hectic in a few weeks. Upon examination post mortem, Mr. Kelly found the lungs in a very diseased state with tubercles on the external surface of the right

The circumstances of the case, however, were different, and the diagnosis was infinitely more easy in the one than in the other. The case is recorded in the Philosophical Transactions for 1823, by Dr. DAVY.—*Med. Gazette.*

---

*History of a Case of Inflammation of the left Spermatic Vein, and Sinuses of the Uterus; with the Appearances on Dissection.* By ROBERT LEE, M.D. &c. Physician to the British Lying-in Hospital.

September 21st, 1827.—Mrs. Somerville, æt. forty, was delivered of her seventh child on the 18th instant, after a natural labour of three hours' duration. Yesterday afternoon she was attacked with a severe rigor, which was speedily followed by acute pain in the hypogastrium and loins, suppression of the lochia, nausea, urgent thirst, and increased heat of skin. In the evening she was delirious, and slightly comatose.

She is now roused with difficulty, and makes no complaint but of pain in the left iliac region. The abdomen is unusually distended, but neither hard nor tense, and pressure produces no uneasiness except between the left ileum and umbilicus. The uterus can still be felt above the brim of the pelvis, large and hard, and very painful on pressure. The milk and lochial discharge are suppressed. The countenance is pale and anxious, respiration hurried; pulse 130, weak and intermitting; tongue white and moist; bowels have been opened by castor oil.

A vein was opened in the arm, but only ten ounces of blood could be procured. Twenty leeches were applied to the hypogastrium, and calomel and antimonial powder were administered every four hours.

During the 22d, the stupor continued to increase, the abdomed was much more distended and painful; the respiration more hurried and laborious; and the pulse extremely quick, feeble, and intermitting. She became completely comatose in the evening, and died on the morning of the 23d.

*Dissection*.—The intestines were slightly distended with gas, but there was no trace of inflammation on any part of their peritoneal surface, and no fluid effused into the sac of the peritoneum. On turning aside the intestines, the left spermatic vein, from the uterus to its junction with the left emulgent vein, was seen distended to nearly the size of the vena cava itself. The cellular membrane surrounding it was highly vascular, and adhered closely to its external coat. On laying open the vein, a dark-coloured, firm coagulum of blood filled it throughout its whole course, but did not adhere to its internal surface, except near its termination, where it was lined with a layer of lymph. The coats of the vein were thicker and firmer than usual, and the internal membrane was of a bright scarlet colour; as was that lining the veins of the uterus, near the fundus on the left side, the part to which the placenta had been attached. The substance of the uterus in this situation was of a dark livid colour, remarkably soft in its texture, and easily torn with the fingers. The corresponding ovary and fallopian tube was also very soft, and of a dark red colour, and shreds of coagulable lymph adhered loosely to their surface. The left renal vein was in the same state as the spermatic, and the substance of the left kidney was soft and vascular. In other respects the abdominal lobe. There was extensive adhesion to the pleura, particularly at the place where the pain had been most felt before the operation. An abscess was found in the right lobe, which contained about four ounces of fetid purulent matter.—EDITORS.

viscera were in a healthy state, and nothing unusual was perceived in those of the thorax. The brain was not examined.

The preceding case, with four others of a similarly malignant character, came under my observation in the two last weeks of September, 1827. As all the individuals so attacked had been attended during labour by the same midwife, and as no example of any febrile or inflammatory complaint of a serious nature occurred among the other patients of the extensive institution under my charge, during the period above mentioned, I was led to conclude that the disease was propagated by contagion.

Inflammation of the veins of the uterus after parturition, has been described by several pathologists;* but the circumstance of the inflammation extending along the continuous membrane of these and the spermatic and renal veins, to the vena cava, has scarcely been noticed,† although it would not appear to be an uncommon occurrence. Dr. D. Davis possesses a preparation, exhibiting the left spermatic and renal veins obstructed with coagulable lymph, which he informed me was taken from a patient who died of puerperal fever a few days after delivery.

Dr. Carsewell, to whom I mentioned the case of Mrs. Somerville, recollects having seen in Paris similar examples of inflammation of the spermatic veins in patients who had fallen victims to puerperal peritonitis. He informed me also that he had occasionally found in the spermatic veins of females advanced in life, concretions and disorganizations of various kinds, obviously the products of inflammation at some remote antecedent periods.—*Ibid.*

---

### SURGERY.

*Phlegmonous Erysipelas cured by Compression.*—Case I. A woman, sixty-five years old, was admitted into the Hospice de l'Ecole, in the last stage of decrepitude. Her leg was swollen, painful, and of a brownish red colour. The pain was increased by pressure, but the colour remained the same. The subcutaneous cellular tissue had a boggy feel. The knee was also much swoln, and there appeared to be a small quantity of fluid in the joint. The inflammation soon extended to the thigh, and there assumed the same character. That the case was one of phlegmonous erysipelas, could not be doubted: the condition of the patient, however, precluded the possibility of abstracting blood with any degree of safety. Compression of the whole limb was ordered, and applied in a very careful manner. Whenever the bandage became loose, it was again applied. By this treatment at first some pain was given, but it quickly subsided, and the extensive inflammation soon terminated by resolution, from the effects of pressure alone.

The state and age of the patient rendered it very probable that gangrene of the affected parts would take place.

Case II.—A man, sixty-five years of age, was attacked with phlegmonous erysipelas of both legs. They were swoln to an enormous size, and the skin was so tense that it was apprehended it would burst. The colour at the surface was a reddish brown, which did not disappear on pressure. The pain was severe. As the patient appeared to be of an apoplectic diathesis, he was

* Dr. J. Clarke's Practical Essays on Pregnancy, &c. pages 63, 72.

† Mr. Wilson, in the Transactions of a Society for the Improvement of Medical and Chirurgical Knowledge, vol. iii. page 65.

twice bled, but no effect was produced upon the inflammatory affection. Pressure was applied by means of bandages, and in six days the parts were restored to a natural appearance.—*Clinique des Hôp.*

---

*Fracture of the Neck of the Thigh-bone.*—Mr. Lizars remarks, in his description of the bones of the lower extremity, that he has been informed, by Professor Dzondi, that he has seen several instances of bony union of the neck of the os femoris when fractured within the joint.

---

*Description of a Case of Fungus Hæmatodes.* By G. Macpherson, Esq.—Many years have elapsed since the disease to which I beg to call the attention of the Society was first accurately described by Mr. J. Burns, of Glasgow, and received from him the name of *spongoid inflammation*, but since the publication on the same subject by Messrs. Hey, Freer, and Wardrop, more generally known by that of *fungus hæmatodes.*

It has been known to attack almost every part of the body; but, so far as I am aware, there are no sure indications from which we can at first ascertain its real nature, except when it occurs in the eye, as the small tumor with which it has been observed to commence in other parts assumes no peculiar form until of some standing, when it has a fluctuating feel, points irregularly at several places, and the surrounding parts become of a deep red colour. The tumor now conveys the idea, from the undulating feel, that it contains matter; and if by mistake cut into, or allowed to take its course and burst, a serous bloody matter is discharged, and soon after a soft, bluish fungus protrudes, which increases very rapidly, and bleeds profusely from the slightest accident; a fluid of a peculiar but most disagreeable odour is discharged, the neighbouring glands swell, and other parts become affected; and ere long, from the discharge and irritation on the constitution, death puts a period to the unfortunate individual's sufferings. The only disease with which it is likely to be mistaken is cancer, and happily the same treatment applies to both: however, there are symptoms by attention to which the two can be readily distinguished. Fungus hæmatodes generally attacks children under twelve years of age. The tumor, before the ulcerative process takes place, has a circumscribed, equal, elastic feel; when it ulcerates, its substance is soft and brain-like, and the fungus shoots out, which increases daily, and never diminishes by ulceration. Cancer, on the contrary, is most commonly met with in subjects advanced in life. The tumor is hard, firm, and inelastic; it is not unfrequently so much diminished by ulceration, and assumes such a deceitful appearance, that one unacquainted with the nature of the disease might be led to hope that it was about to cicatrize.

After the appearance of the fungus, when the character of the complaint is fairly developed, the only hope that remains of preserving life is by attempting to eradicate the disease with the knife; but, from the circumstance of the pain and inconvenience not being excessive at this stage of the disorder, it is with difficulty that the patient, if an adult, or the parent of a child, can be convinced of the immediate necessity of having the mass extirpated, or the limb amputated, and they are naturally very anxious first to try the effect of palliative measures. It, however, becomes the imperative duty of the medical attendant to use every endeavour and persuasion in his power to induce a submission to the operation, at the only time its performance is likely to be attended with success; for it is a melancholy fact that there is no chance of any amendment taking place, either spontaneously or from the use of remedies; and there are but few instances on record where cures have been the result of operations, if not had recourse to at an early stage of the disease.

I have now the honour of submitting to the Society particulars of the only case of fungus hæmatodes of the eye that has come under my observation in this country, which I think will be considered interesting, as the result has been so much more favorable than was to be expected, from the circumstance of the organ not having been extirpated till the disease had proceeded so far as to render the chance of success nearly hopeless.

On the 15th of July, 1825, a boy named Toofauny, about three years of age, was brought to me with fungus hæmatodes of the eye, in a very advanced stage of the disease. His mother, who accompanied him, informed me that neither herself nor her husband were aware that any thing serious ailed the child, (although he was very fretful, and sometimes feverish,) until the middle of March last, when they accidentally discovered that he had lost the sight of his left eye, and then they were led to examine it; but all that they or their neighbours could observe was a slight inflammation, of which the other appeared to be free. A Kubraj, or village native doctor, was sent for, who stated that the inflammation was occasioned by some sand having got into the eye, which he extracted, or pretended to extract, and then took his departure, assuring them that the child would recover his eyesight in the course of two or three days. Some weeks subsequent to this, the child complained of violent pains in his head; had very disturbed sleep, from which he frequently awoke with a scream; his appetite was very uncertain, some days hardly tasting his food, and at other times being almost insatiable.

These symptoms continued, with little variation, for nearly a month; and, according to the woman's account, the diseased eye, although it became internally of a yellowish colour, neither assumed any irregular figure nor protruded from the orbit, until a few days before it burst, when it became much enlarged, and of a blackish hue. The relief which the poor little sufferer experienced from the bursting of the organ was so great and so immediate, that the parents were led to entertain sanguine hopes that now all bad symptoms would disappear; and these hopes were for some days strengthened by the following circumstances: he complained of no pain in the head, and of very little in the eye; his appetite became more regular, and he seemed to relish his food; he slept soundly, although only for short periods, and awoke apparently refreshed. These delusive appearances, however, were of but short continuance, as the fungus began to protrude beyond the orbit about the middle of the month of May, and a day or two afterwards bled so profusely from a very slight accident, as to cause the greatest alarm to the parents. It continued daily to increase, and occasionally to bleed, from this period till the 10th July, when they despaired of the child's recovering; but, as a last recourse, determined on bringing him to a medical man; a mode of conduct which, it may not be amiss here to remark, is too frequently adopted by the natives of this country.

When brought to me, the poor boy presented as unseemly an appearance as I had ever witnessed. The left cheek was nearly covered with the fungus, which was of a globular form. One side had a blue tinge, while the other was of a dark red colour. It adhered to both eyelids, excepting for a small distance at the external angle of the superior one. He was so weakened and emaciated as to be unable to sit up without support. He seldom spoke, but whined and cried much. The light seemed to give him great annoyance: his head was therefore kept covered with a cloth, both to exclude it and keep the flies off, which were very troublesome. The glands about the parotid and under the jaw were swollen, but the integuments not at all discoloured; and he experienced little or no pain from pressure being made on them.

It was evident that death must soon ensue, if something were not speedily done. I was consequently induced to recommend the extirpation of the eye, although I confess my expectations of its being of service were any thing but sanguine. I explained to the mother that the disease had been allowed to proceed to such a pitch, that there was scarcely a hope of the operation being attended with success, but that it was the only chance there remained of preserving the life of her son. The poor woman was convinced of the urgency of the case; I therefore determined on operating the succeeding morning.

I commenced the operation at the outer angle, where the tumor did not adhere to the palpebræ; but, as they were both diseased, I thought it advisable to remove them along with the fungus, instead of first destroying the adhesions, then extirpating the eye, and afterwards cutting away the lids. On making my first incision, an alarming hemorrhage took place, when the child fainted, which had the effect of checking the bleeding, and the rest of the operation was got through with a common scalpel in two or three minutes, without any occurrence worthy of remark. The inside of the orbit was now carefully examined, and every thing of a suspicious appearance removed with the knife. The bleeding was very trifling. I therefore thought it unnecessary to fill the orbit with lint, as is usually done, and merely applied a compress and bandage. An anodyne draught was now given, from the effects of which he slept nearly twelve hours.

17th July, nine A.M.—Complains very much of vertigo and pain in his head. Has had no stool since yesterday morning. Habeat Pulveris Jalapæ gr. x.; Submuriatis Hydrargyri gr. iij. stat. sumend.

Four P.M.—No stool; symptoms much the same; excruciating pain in the head.—Loosened the bandage. Injiciantur quamprimum per anum enematis domestici unciæ octo.

Nine P.M.—Has had two fetid and dark-coloured stools. Pain in the head less severe.—Repetatur Pulvis.

18th.—Two copious but very fetid evacuations during the pain: pain in the head much relieved; countenance more lively; pulse quick; tongue foul, and surface rather heated; no inclination for food.—Repetatur Pulvis.

19th.—Passed a good night. Has had three copious stools since yesterday, of a more healthy appearance. Pain in the head almost gone; pulse calm; tongue cleaner; and surface moist and cool.—Vespere repetatur Pulvis.

20th.—Much improved in appearance. Bowels well opened; headach and all febrile symptoms gone. Took some boiled rice and milk, which he seemed to relish much.

From this period I commenced giving him small doses of bark, two or three times a day, taking care to keep his bowels rather loose. This practice was attended with the most beneficial effects, as his health and strength daily increased, and the wound occasioned by the removal of the tumor healed kindly by granulations in less than three weeks; after which, he and his mother left this for their home, with a supply of bark, and some purgative powders to be given occasionally.

I saw the boy last April, nine months subsequent to the operation, when he was in perfect health. The swollen glands had not at all increased in size; neither had he felt any pain in the orbit since he left this. I have not seen him since; but my native doctor tells me he had a severe fever last month, from which he recovered, and was, when he last heard, quite well.

The fungus (which I now send) weighed, the day after it was extracted, seven ounces, five drachms, one scruple; and was so soft and pulpy, that I was under the necessity of steeping it in acid, with a view to harden and preserve it.—*Trans. of the Med. and Phys. Society of Calcutta.*

